# Staying Active While Staying Home: The Use of Physical Activity Technologies During Life Disruptions

**DOI:** 10.3389/fdgth.2021.753115

**Published:** 2021-10-28

**Authors:** Joseph W. Newbold, Anna Rudnicka, Anna Cox

**Affiliations:** ^1^NorSC, Computer and Information Sciences, Northumbria University, Newcastle upon Tyne, United Kingdom; ^2^University College London (Interaction Centre); (UCLIC), University College London, London, United Kingdom

**Keywords:** COVID-19, physical activity, activity tracking, virtual coaching, life disruptions

## Abstract

One impact of the Covid-19 lockdowns was a restriction on people's ability to engage in physical activity in previously routine ways. This paper presents a two-stage mixed-method study exploring how people used technology to stay physically active during this period. We found that activity trackers reminded people to be active, while virtual coaching (i.e., video tutorials and online classes) helped them stay connected. The lockdown increased people's awareness of their activity levels and removed barriers to exercise, for example by giving them greater control over their time. However, it also created new challenges, with lack of time and space, injuries due to sudden changes in activity, and anxiety around lockdown, putting limits on physical activity. We highlight future directions that must be addressed to maximise the benefits of physical activity technologies for people trying to stay active during major life disruptions.

## Introduction

Physical inactivity is an ongoing global health concern. Regular physical activity has the potential to prevent 46% of deaths related to inactivity (1). It has also been shown to strengthen our immune system as we age (2), lower risk of heart disease, high blood pressure and diabetes (3), as well as dementia, various cancers and strokes, while improving mental well-being and reducing anxiety and depression (4). This demonstrates the potential for physical activity to support healthy living. However, despite this, many people struggle to maintain a regular physical activity routine, due to a range of individual, social, environmental, and psychological factors (5, 6). While previous works have demonstrated the potential for technology to support people staying active (7), creating lasting behaviour change with physical activity technology still proves difficult with issues of abandonment common (8, 9). Similarly, many of the physical activity technologies available are not designed with usability and behaviour change in mind (10), with many apps lacking theoretically backed content (11).

However, in the context of the COVID-19 crisis, further concerns arose around physical activity. With many countries instigating stay at home policies (dubbed lockdown in the UK) to prevent the spread of the virus, there were concerns regarding the impact this could have on people's opportunities to undertake physical activity (12, 13). In turn, this came with recommendations to stay active and the allowance, for those able, to break lockdown for the purposes of physical activity (14, 15). As prior studies show, these kinds of disruptions can prove detrimental to people's physical activity routines, with environmental factors being one of the main determinants of activity levels (16). Hence there is a need for supporting people with staying active during this time. Moreover, this period gives a unique lens to explore how people respond to significant life disruptions and understand how we may better support them. In this paper, we define “life disruptions” as a significant disruptions of ones circumstance, lifestyle or routine. These have been explored in previous works with regards to personal; distress, changing health and grieving (17, 18), however such insights may also be beneficial to other situations for example, moving to a new environment or a change in work or family commitments.

Massimi *et al.*, highlight the significance of technology in the way we respond to life disruptions and how it can help foster social ties as well as create a grounding for us during times of uncertainty (17). Lacovides and Mekler's work also demonstrates how people use gaming to support them through times of turmoil (18); in their study, video games were used as a way of regulating emotions and supporting socialisation during difficult life experiences. The question remains then, if such technology can be used to support people during life disruptions, can physical activity technology offer the same support during a time of global crisis where people's physical activity routines are at risk. If so, how will people leverage such technology during this time to stay active?

In this paper, we present a two-stage study which examines how people who were already fairly active used technology to maintain their physical activity in the face of this major life disruption. Firstly, an initial survey-based study looks at how people (*n* = 390) tried different technologies and activities to stay active during lockdown, and the benefits and barriers that they experienced. Secondly, a series of four follow-up surveys examine how people's (*n* = 126) use changed over time. From these studies this paper offers three main contributions:

1. An overview of activity levels and physical activity technology use during lockdown
2. An exploration of the benefits and issues that people faced while using technology to stay active
3. Directions for future development of how technology can help us stay active during life disruptions.

## Background

### Physical Activity Technology

The benefits of physical activity are widely known; physical activity improves cardiovascular health, mental health and helps reduce the risk of many other major health concerns (1, 3, 19). However, it is difficult for individuals to maintain regular physical activity due to a variety of environmental factors such as lack of space or time for physical activity, or motivational factors (5, 6). Overcoming these barriers has long been a topic of interest for HCI researchers and we have seen many technologies designed to support people in staying physically active. Previous research has shown the effectiveness of activity trackers in helping to improve awareness and reflection (20), as well as in supporting goal setting and developing a routine (21, 22). Automated coaching has been shown to be an effective way for people to develop a new exercise routine (23). Moreover, exergames have been shown to help make exercising at home more convenient, provide instruction and make exercise more fun (24, 25). In addition, motivational technologies have been designed to provide rewards/incentives and make use of behaviour change theory to help people develop routines (26–28).

However, we also know of numerous ongoing issues within exercise technology as it is used in the real world. Many of the fitness apps readily available to the public lack a user-centred design approach as well as having designs with little theoretical basis (10, 11). These apps often fail to reflect the design considerations needed to create effective activity technologies such as individual factors, e.g., ability and motivations, and providing appropriate awareness and incentives (29). For example, Rutjes et al., report a set of studies where physical activity practitioners set out how e-coaching lacks key elements that are present in in-person sessions (30). They highlighted the importance of the social aspects of coaching, adjusting to contextual information provided by the individual and facilitating ongoing motivation. In addition, there are a set of barriers to habit formation, that are difficult to solve through technology alone (31), and with fitness technologies, abandonment and disengagement are common problems (8, 9, 32). We were therefore, interested to know whether, in a situation such as a national lockdown when people lose their preferred exercise location and the social aspects of exercise, whether technology might be able to help bridge the gap.

### Physical Activity in Lockdown

Among the many challenges the world faced with the COVID-19 pandemic, the impact of lockdown on people's physical activity could present a more hidden danger. Initial research warned of the potential impact that lockdown could have on inactivity levels and the knock-on effects it would have on cardiovascular health and obesity (12, 33). Hall et al. describe it as a “tale of two pandemics,” with the obvious concerns surrounding COVID-19 and reducing the spread of the virus, paired with the ongoing concern of global inactivity and sedentary behaviour (13). They call for aggressive efforts to be made to combat the effect that lockdown measures have had on people's activity levels. Indeed, Ammar et al. surveyed people across Europe, North-Africa, Western Asia and the Americas, and reported decreases across activity levels (vigorous, moderate and travelling activities such as walking) as well as an increase in sitting time (34). Moreover, Ranasinghe et al., recommend regular physical activity as a key measure to help deal with the mental health strains associated with isolation and also to maintain a strong immune response for battling the virus itself (35).

Prior work shows how environmental factors can disrupt routines and lead to inactivity (36), with access to space, for example, being a strong determinant of physical activity. Disruption of these routines has been observed during the pandemic. A longitudinal study of Americans' activity and mental health over the course of the pandemic demonstrated how the pandemic disrupted people's routines and led to a reduction in step counts from 10,000 to 4,600 a day in some populations (37).

These issues have led to a series of suggestions as to how technology could be the key to overcoming these new barriers to physical activity. These included, for instance, recommendations for policy surrounding digital health technologies to support innovation in the wake of the pandemic (38) as well as recommendations for how technology may support people coping with the pandemic (39), or how VR technology can support remote delivery of physical activity instruction (40). Moreover, while initial data provided by physical activity technology companies such as Garmin and Fitbit suggest that there was an overall decline in the activity levels of their users at the start of lockdown, these companies started to see activity levels increasing again over time (41, 42). Similarly, EE (a mobile telephone network) reported increased use of Strava during lockdown, an activity tracking app on their network, during the first months of lockdown (43). Marchant el al. examined the impact of eHealth technologies on activity levels and motivations (44). In a quantitative study of people in confinement during the pandemic in France, they found significant effects of eHealth technologies on a number of psychological constructs associated with physical activity and reported activity levels. This work highlights the potential that technology may have in helping people remain active during lockdown. However, it is unclear how the issues we already know about, surrounding the use of technology to support physical activity, will play into people's ongoing use during these life disruptions and which aspects of physical activity technology design will impact people's experience.

## How Did People Use Technology to Stay Active During Lockdown

This two-stage study explores people's experiences of different types of technology to support physical activity during the crisis. Overall, we examine two aspects of this problem: (1) the motivations and uptake of new technology in response to lockdown and (2) how that use, and motivation, changed over time. Therefore, the study aims to explore people's experience with physical activity during lockdown, understand what did/did not work and from this, determine what lessons can be taken forward into the design of new technology. These aspects are covered by our research questions:

- How did activity levels and technology use across lockdown change?
- How did people experience the impact of lockdown on their physical activity?

To address these questions, we deployed a number of mixed-methods surveys, to look at both quantitative data pertaining to peoples technology use and activity levels during lockdown as well as qualitative experiential data pertaining to how people perceived the benefits that technology had on their staying active during lockdown. The study took place in two stages, firstly an initial survey to understand the different technologies people had tried during lockdown and the initial impact lockdown had on their physical activity. Secondly, we deployed four follow-up surveys to examine people's ongoing activity levels and physical activity technology use. These studies together offer a snapshot of people's experience of staying active in lockdown and what we can learn from it.

### Participants

We recruited participants through social media accounts (Twitter and Reddit), through word of mouth, and university-wide newsletters. Participation was open to individuals over the age of 18. Survey respondents were entered into a prize draw for 10 x £20 amazon vouchers for the initial survey and then a £50 amazon voucher prize draw, at each follow-up stage.

The initial survey was launched at the beginning of May 2020 and remained open until the end of May. As participants joined the study, and responded to follow-up requests, at different points in time, the final follow-up survey was open until the 12th of July. This was to allow participants who started the initial study at the end of the month to fully complete the study (four weeks plus some slack from their response times to the follow ups)

In the UK, a country-wide lockdown was announced on the 23rd of March 2020. People were asked to only leave the house when shopping for basic necessities as infrequently as possible and for one form (maximum 20 min) of exercise per day. Parks and leisure centres were closed. Leaving home was also permitted when seeking medical help, providing help to a vulnerable person or travelling for work if absolutely necessary. Changes to lockdown were introduced on the 10th of May 2020, when people could start exercising outside more than once a day and could also go to the park. On the 4th of July, there were further relaxations to lockdown measures, which allowed people to leave their house multiple times a day for reasons other than exercise. Gyms and pools remained closed until the 25th of July.

#### Initial Survey Sample

A total of 617 people initiated the survey, by agreeing to proceed in response to the initial consent question, and *n* = 223 dropped out over the course of the survey, resulting in a sample of *n* = 394 participants. We then eliminated *n* = 2 duplicate responses and *n* = 2 responses from participants under the age of 18. The final sample consisted of 390 participants who completed the full initial survey, with a mean age of 36 (SD = 12). The participants reported their gender as follows: *n* = 297 female, *n* = 82 male, *n* = 4 non-binary, and *n* = 7 preferred to self-describe.

#### Follow up Survey Sample

Out of the initial survey sample of *n* = 390, a total of *n* = 126 participants completed all 4 weekly follow-up surveys. The mean age of these participants was 36 (SD = 11), with participants reporting their gender as follows: *n* = 101 female, *n* = 20 male, *n* = 1 non-binary, and *n* = 4 preferred to self-describe.

### Materials

The initial survey consisted of two parts. The first was a measure of the individuals' physical activity levels using the WHO's Global Physical Activity Questionnaire (GPAQ) (45). Participants were asked to fill in this questionnaire retrospectively for their activity levels before lockdown and during lockdown. The second part was a set of questions around what technologies have been used combined with open text fields asking:

- “Please provide some examples of technology you have used to support your physical activity”
- “What do you think the benefits of such technologies are in supporting your physical activity?”
- “How do you think the COVID-19 crisis has impacted your physical activity and your use of technology?”
- “Tell us a bit about the types of physical activity you used to engage in before the lockdown period and how that has changed”
- “How has your motivation to engage in physical activity changed due to the lockdown?”

These questions aimed to probe deeper into the how and why of people's physical activity and physical activity technology use.

Each of the four follow-up surveys asked participants to fill in the WHO's Global Physical Activity Questionnaire for the week as well as report what activities/technologies they used that week. There were also open questions where participants were asked to describe the benefits they had perceived that week and any issues they had faced. All surveys were hosted on the online Qualtrics platform.

### Design

The design of this study had two aims, to investigate the impact of technology on activity levels across lockdown and to explore how people experienced physical activity technology across lockdown.

The first of these research aims is addressed through our quantitative measures, with the dependent variables coming from the GPAQ as a measure of activity levels (operationalised by recording how many minutes per week people spent on each activity) and physical activity technology use (activity tracking, fitness planning, online coaching etc.). These measures were taken in the initial survey (for both before and during lockdown) and then weekly in the follow-up surveys.

To address the second research aim, we asked the participants to describe their experience of various aspects of physical activity technology. These responses were the focus of the qualitative analysis reported in this paper, which is used to both unpick aspects of the quantitative findings as well as give an idea of people's broader experience of staying active during lockdown which will help us build an understanding of the role technology plays.

### Procedure

#### Administration of Surveys

The study consisted of two stages. Firstly, we conducted an initial survey, scoping people's demographic information, physical activity levels, and physical activity technology use. We then conducted weekly follow-up surveys for a period of 4 weeks, monitoring people's on-going physical activity and what, if any, interventions they have been trying. To facilitate the follow-up surveys, an automated email with a link to the next week's follow-up was sent 7 days after the previous survey was completed. Failing completion, a reminder email was sent after 2 days. Participants were free to withdraw from the follow-up surveys by simply skipping 1 week and would then, following one reminder, not be contacted again.

## Results

### Quantitative Analysis

The analysis of the survey data was done in two parts, an analysis of the quantitative data including the GPAQ and responses on physical activity technology use, which is used to gain an understanding of our sample's activity and technology use before and across lockdown and a more in-depth qualitative analysis of people's free-text responses around the perceived benefits of technology and their experience of staying active during lockdown. All 390 participants were submitted to analysis for the initial survey. However, for the follow-up surveys, only participants who completed all five surveys (*n* = 126) were included in the analysis.

The quantitative data analysis was conducted in IBM SPSS Statistics for Macintosh, Version 25.0. The GPAQ data was cleaned according to the instructions outlined in the GPAQ Analysis Guide (45): values 15,30,45, and 60 in the hours' column were moved into the corresponding minutes variable if the minute variable was empty or zero (assumed data recording error); missing values were converted to zero and hours and minutes were added to arrive at a total time per day; where total minutes per day exceeded 16 h for any activity or 24 h for sitting, the participant was removed from all analyses; where values were inconsistent (participant reported 0 days but 0 < total min per day for any activity), the participant was removed from all analyses. This resulted in a final sample of *n* = 333 participants who completed the full initial survey, and a final sample of *n* = 84 participants who completed all follow-up surveys, whose data were suitable for quantitative analysis.

By multiplying the indication of how many days per week the activity was undertaken by total daily minutes, we arrived at total weekly minutes per each category. Sitting minutes per day were multiplied by seven, as no question about days per week was asked. In the initial survey, quantitative data were collected about participants' levels of activity before and during the lockdown.

### Qualitative Analysis

Qualitative data was subjected to a thematic analysis as outlined by Braun and Clark (46). In the illustrative quotes used in the results section, the initial survey respondents are represented by “**P#**” whereas follow-up responses are indicated by “**FP#**.” While this means that some participants may be referred to by two separate indicators, the authors felt that this would be the clearest representation of the two samples. For the qualitative analysis, all responses were collected into Nvivo. From this, familiarisation with the initial survey dataset was conducted and then through an initial coding of responses for questions on, the physical activity technology use reported, the benefits of using technology, people's motivation for staying active and finally the perceived impact of the lockdown on their use. From these coded responses initial themes were constructed, which were then reviewed and then brought together across questions to create the main themes for the initial survey of physical activity technology use and benefits and the impact of lockdown on motivation and physical activity technology use.

For the follow-up surveys, responses from each participant to questions on physical activity technology use, perceived benefits and issues faced were connected across the surveys. These group responses were then coded for changes in responses over time and overall response, for example, “Baseline: Keeps a record of what I do. Follow up 1: Tracks. Follow up 2: Prompts me and gives guidance. Follow up 3: monitors steps so I don't have to think about it. Follow up 4: Sleep monitoring” **FP5**, would be coded for both the “continued use of self-tracking” and “use technology to be accountable.” These codes were then used to construct themes that reflect the overall responses across the follow-ups, rather than looking at individual points in the follow-ups, to get a clearer picture of people's journey throughout lockdown.

The results are presented below, divided by research questions. Results are presented chronologically: first, results from the initial survey, showing the initial impact of lockdown and then the follow-up surveys, showing the ongoing impacts. At each stage, we firstly examine people's physical activity during lockdown and how technology was used to support physical activity and then how people perceived the impact of lockdown on their exercise and the role of technology.

### How Did Activity Levels and Technology Use Across Lockdown?

In this section, examine people's activity levels as measured through the GPAQ for different classifications of activity, firstly at the start of lockdown and then across the four follow-ups. Then, we examine how people used technology to support their physical activity across lockdown.

#### How Did Activity Levels Change at the Start and Across Lockdown?

Table 1 summarises activity levels reported for two time periods: before and during the lockdown, for the sample of participants who completed the initial survey and whose data were suitable for quantitative analysis (*n* = 333). Paired *t*-tests suggested that the lockdown did not influence the time spent on vigorous work activity. Participants experienced a decrease in time spent on moderate work activity and travel during the lockdown, and an increase in time spent on moderate and vigorous sport and sitting time.

**Table 1:** Mean total reported min per week across activities (vigorous work, moderate work, travel, vigorous sport, moderate sport) and sitting in the initial survey (*n* = 333), before and during the lockdown.

	**Mean reported min (SD) per week**					
	**Vigorous work** **activity**	**Moderate work** **activity**	**Travel**	**Vigorous sport** **activity**	**Moderate sport** **activity**	**Sitting**
Before the lockdown	50 (225)	186 (389)	427 (459)	175 (274)	192 (361)	3,388 (1,343)
During the lockdown	34 (167)	93 (344)	158 (302)	223 (423)	254 (445)	3,908 (1,632)
Paired *t*-tests (significant results in bold)	(*t*_(332)_ = 1.227, *p* = 0.221)	**[*****t***_**(332)**_ **= 4.504**, ***p*** **= 0.000]**	**[*****t***_**(332)**_ **= 9.878**, ***p*** **= 0.000]**	**[*****t***_**(332)**_ **= −2.103**, ***p*** **= 0.036]**	**[*****t***_**(332)**_ **= −2.548**, ***p*** **= 0.011]**	**[*****t***_**(332)**_ **= −6.772**, ***p*** **= 0.000]**

**Figure 1:**
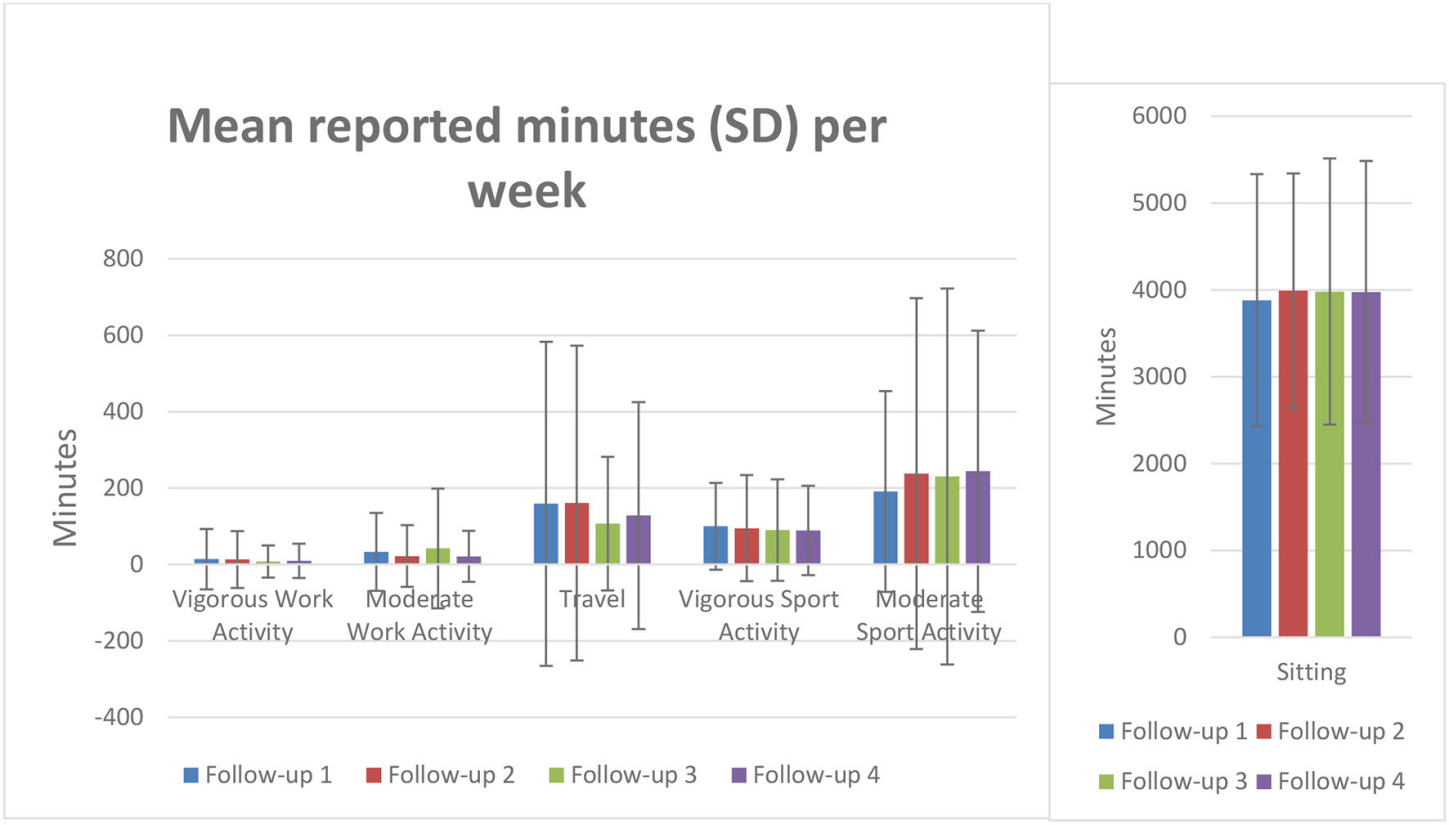
The reported minutes of exercise across the follow up surveys.

Table 2 and Figure 1 summarises activity levels reported for four follow-up stages, for the sample of participants who completed all follow-up surveys and whose data were suitable for quantitative analysis (*n* = 84). A series of repeated measures ANOVA tests (a Greenhouse-Geisser correction was required for all categories except for “vigorous sport”) were performed to compare physical activity across the four follow-up stages. These results suggest that, for participants who remained within the study, mean activity levels across follow-up stages were stable.

**Table 2:** Mean total reported minutes per week across activities (vigorous work, moderate work, travel, vigorous sport, moderate sport) and sitting in the four follow-up surveys (*n* = 84).

	**Mean reported min (SD) per week**					
	**Vigorous work** **activity**	**Moderate work** **activity**	**Travel**	**Vigorous sport** **activity**	**Moderate sport** **activity**	**Sitting**
ANOVA results	*F*_(1.083, 89.903)_ = 1.3001, *p* = 0.261	*F*_(1.665, 138.182)_ = 1.525, *p* = 0.223	*F*_(2.150, 178.419)_ = 0.708, *p* = 0.504	*F*_(3, 249)_ = 0.303, *p* = 0.823	*F*_(1.677, 139.179)_ = 0.794, *p* = 0.434)	*F*_(2.532, 210.153)_ = 0.246, *p* = 0.832

#### How Did People's Physical Activity Technology Use Change Across Lockdown?

**Figure 2:**
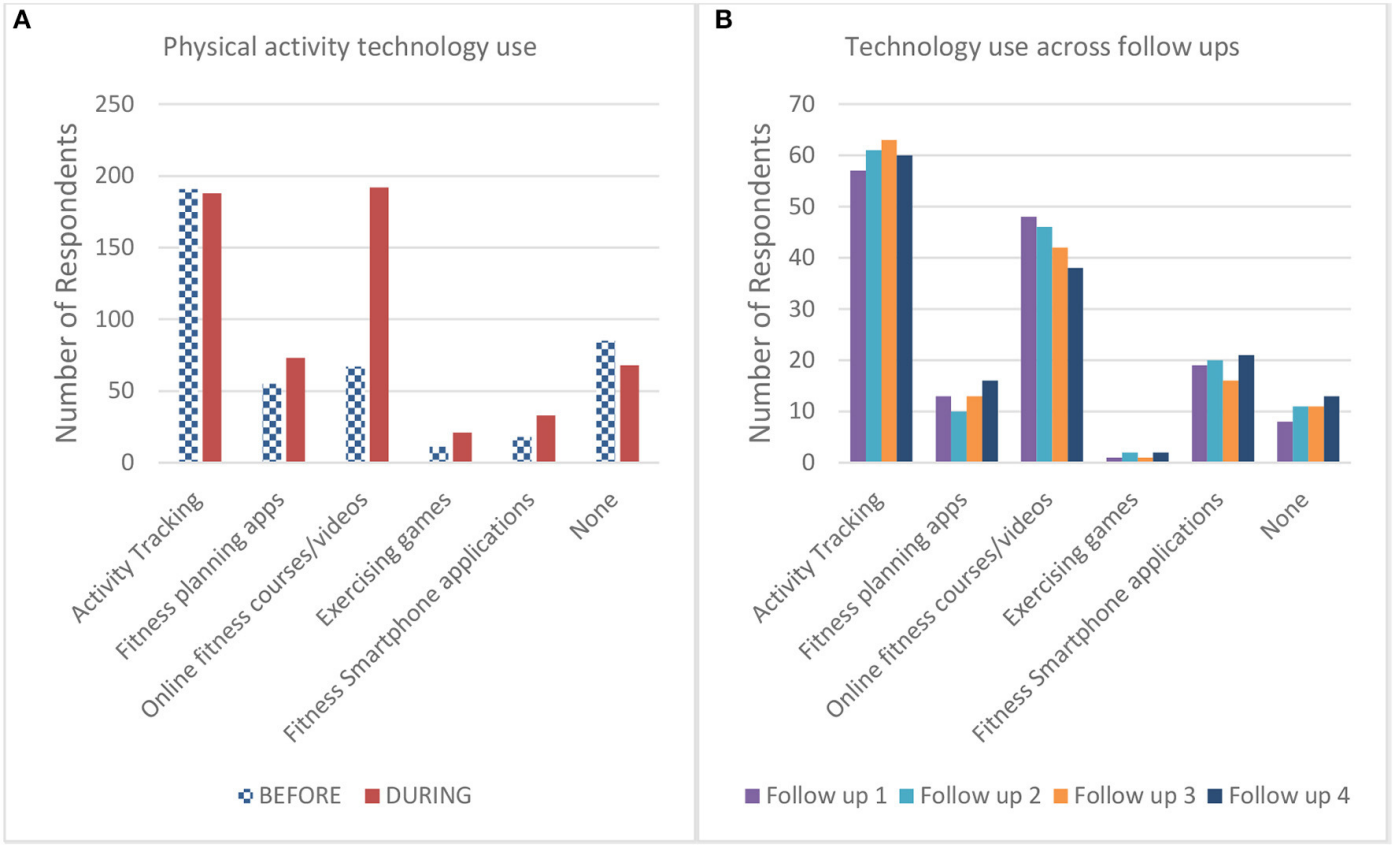
**(A,B)** shows the reported physical activity technology use for participants in the follow-up surveys.

Figure 2 summarises the reported use of technology supporting physical activity before and during the lockdown. Responses were submitted to a McNemar's test to assess the changes in physical activity technology use. Online fitness courses (including virtual training and YouTube tutorials etc.) saw a significant increase (*p* < 0.001), as did the use of smartphone fitness apps (*p* < 0.001) and overall use of physical activity technology (i.e., use of any technology) saw a significant increase (*p* < 0.001). Figure 2B summarises the reported use of technology supporting physical activity throughout the lockdown from our follow-up sample. Responses were again submitted to a series of McNemar's tests comparing between each follow-up with no significant changes in uptake found.

### How Did People Experience the Impact of Lockdown on Their Physical Activity?

In this section, firstly we outline the qualitative response participants gave on the benefits they perceived from using different technologies and how those perceived benefits change over time. Secondly, we examine how lockdown had changed their motivation around staying active.

#### What Were the Perceived Benefits of Using Physical Activity Technology During Lockdown?

##### Activity Tracking

One of the most common technologies participants discussed was activity tracking (*n* = 98). This came in the form of not only dedicated trackers such as Fitbits or Garmin watches but also more general smartwatches or built-in step counting on individuals' smartphones. Such technology was seen to support record-keeping and monitoring progress (*n* = 88). Participants noted that being able to see their activity levels was motivating both as they desired to maintain a certain level day-to-day and also in helping them set goals to work towards. The act of recording an activity itself helped with the actualisation of feeling active (*n* = 71).

“*Encourages me to beat personal bests and so on. If I go running and don't record it, it somehow doesn't feel as worthwhile. It is satisfying to look at your stats afterwards”* **P106**

Participants also noted that tracking their overall activity served as a reminder to move. This was used in addition to the inactivity notifications to help combat sedentariness due to remote work (*n* = 23). Some participants specifically noted that their use of technology was to help them track the change in their activity levels during lockdown (*n* = 44). With the lack of commuting and workplace activity, people were worried about their general levels of activity throughout the day and used their activity tracking devices (for example, wearable or smartphone-based) to quantify this change and compare with their previous activity. This was simultaneously seen as a motivating factor to some, but also demotivating for others who found their lack of activity destressing and in turn led to disengagement.

“*I don't look at my iPhone's health app as much, as I know my steps are much lower.”* **P13**

##### Fitness Apps

A number of fitness apps were also used during lockdown (*n* = 98). These ranged from activities specific apps such as DownDog for Yoga as well as more general fitness apps such as MyFitnessPal or the native health apps on smartphones (Samsung health/Apple health). The use of such apps was motivated both to track one's activity and to gain expertise, but also as a way to create accountability. By having a dedicated program/preselected exercise regime, participants felt more inclined to engage in regular physical activity. This structure was said to be very valuable in maintaining ongoing motivation to stay active as well as providing achievable goals (*n* = 44).

“*Keeps me to a regular schedule, helps me track my activity, plans my workouts so I know I'm doing recommended activities and don't have to work out what to do myself.”* **P312**

Running apps were also common among participants, including dedicated training apps such as Couch to 5K and more tracking related apps such as Strava (*n* = 31). Training apps were seen as supportive for those who had recently started running and the predetermined structure helped them to feel accountable. However, more dedicated running apps were viewed more like an extension of activity trackers, to provide more detailed recording and analysis of ones runs. This allowed users to better see their progress. The social aspects of such apps allowed people to share their activity with friends, who provided not only encouragement but additional accountability to continue being active (*n* = 43).

“*I also love the Strava app, as I can easily see how much I have been running/walking and the analysis feature is a great tool to see improvements in time/pace, which in turn motivates me to work hard. I love the social aspect of this app as you are able to follow friends and sporting groups, such as my football club, and really support and celebrate others' achievements. It gives me a buzz when I know I've run a PR or a good time and others acknowledge this too!”* **P289**

##### Virtual Training

One of the technologies that saw the greatest increase in use was virtual training in the form of online classes (*n* = 69) and video tutorials (*n* = 103). However, while for some this seemed to be a replacement for in-person activities, for others it was an entirely new endeavour. People found that virtual training, be that online classes or video tutorials, allowed them to learn new exercises and perform movements safely. With in-person training removed, online content was the only way people had to gain guidance on how to perform certain exercises (*n* = 52).

“*Online videos help me target things i don't know how to do alone and give me more confidence in doing yoga/ barre.”* P61

Others found that the structure and variety in online courses helped them feel motivated, as they would not have to design their own exercise program. This allowed people to not only continue with exercises they were previously doing but gave easy access to new kinds of activity, that they could try out with relative ease (*n* = 24).

“*Give me ideas for new exercises, help me do the exercises correctly, inspire me to work out a different muscle group, give me inspiration and get me off the couch.”* P36

Similarly, virtual group classes offered a way to stay connected with people during lockdown. This also allowed people to continue with exercise classes they would previously attend in person and provided a sense of community (*n* = 29). It allowed not only for socialisation and staying in touch with friends but added back the social commitment people used to motivate them to exercise, giving people some “accountability” (*n* = 43).

“*The livestream classes that allow you to workout with other people have been the best for me personally because I am used to group fitness classes and struggle to get motivated and workout on my own.”* **P338**

##### Accessibility and the Best Available

An overarching benefit that participants saw from the use of technology to support physical activity technology was their accessibility, flexibility and as it was the best they could get in the current situation (*n* = 69). Participants noted that having such easy access to technologies helped them adjust to staying active in lockdown. The fact that smartphones and, to an extent, smartwatches have become more ubiquitous, in addition to the number of free apps and content available online, meant that it was easy for people to transition their activity into lockdown.

“*Also, the training can be followed anywhere at anytime so it's much easier to get motivated and be diligent.”* **P28**

This was also reflected in how people used different technologies together, with the majority of participants using a number of technologies in tandem (*n* = 179). This accessibility was seen to remove some of the barriers to being active, with no need to travel or get specific equipment to be active, physical activity could now be done “rain or shine at home.” Similarly, this accessibility allowed for more flexible scheduling of physical activity (*n* = 18). This was seen as particularly important during lockdown when people were now juggling working remotely, childcare and other concerns all at the same time. The easy access and flexibility of technology allowed them to achieve some balance.

“*I can exercise at home while keeping an eye on my toddler.”* **P234**

In addition, many participants noted that through technology they were able to get the best they could without access to their normal activity (*n* = 29). However, while some said they would prefer to be able to return to their prior activities (in the gym or group sport activities), others noted that the change made it a lot easier for them to maintain regular physical activity. The benefits of participating in exercise during lockdown were said to be not having to travel, not needing specialist equipment or instruction and not feeling self-conscious in a group activity or class.

“*Gyms near me always sell out of spaces for workout classes. If I am able to get a space they are packed and I feel more self-conscious in front of others*.

*The app I use is free and doesn't even have adverts.”* **P278**

##### Changing Benefits Across Follow-Ups

The overarching benefits to using technology across our follow-ups fall into two main factors: staying accountable (*n* = 52) and developing a routine (*n* = 42). Using technology to stay accountable was true across a number of technologies, from having a prescheduled online class to having a specific activity planning app such as Couch 2 5K. However, the most common change from the initial use was seen in activity trackers (*n* = 29), where initially they had been used to track activity and set goals, across the follow-up weeks they became more of a reminder to be active, once people had learned what their desired activity level was. This learning helped people develop accountability with themselves and build a routine around the activity levels they aimed for.

From Follow-up 2: “*It provides accountability for my physical activity and allows me to see improvements. This in turn helps keep me motivated and has allowed me to develop a routine.”* **FP8**

In addition to creating accountability, people used technology to build a personalised exercise routine. Through seeking out instructional videos and goal setting programs, they developed a knowledge base on how to exercise safely. However, many continued to use such instructional videos/classes and became more focused on variety and motivation than learning specific exercises. A key benefit seen was that predefined workouts would be both safe and enjoyable, as well as not having to spend time to create one's own routine.

From Follow-up 3: “*I like having a class to follow or to participate in, to give some variety to my workouts. I find the community/social elements really encouraging, e.g., taking part in a fitness class online with others.”* **FP83**

#### What Were the Changes in People's Motivations Due to Lockdown?

Lockdown had a mixed effect on people's motivation to be active, with some feeling more motivated, some feeling less and for some their motivation remained the same, but other barriers kept them from engaging in regular physical activity. For those whose motivation increased due to lockdown, their reasons included ranged from being more conscious of inactivity, a desire to go outside and mental health benefits.

##### Increased Awareness

Participants noted that they had become more aware of the need to be active because of lockdown (*n* = 113). For some, it was because they had lost their commute and daily activity and they were conscious of the sedentary time, which made them feel that they needed to be more active. Others found that government guidelines around being active had made them more aware of the importance of regular physical activity, while some linked their motivation directly to combating the virus itself. People were also aware they had been eating/drinking more in lockdown and saw exercise as a way to combat this, with some going further to say they felt obligated to come out of lockdown fitter.

“*I feel it more as a necessity not to come out completely out of shape after the lockdown. This kind of necessity motivates me.”* **P23**

##### Time Outside and Mental Well-Being

Others were motivated by a desire to spend time outside, as at the start of lockdown physical activity was one of the few ways people could spend time outside. This desire was fueled by a wish to spend time out of the house, a desire for some alone time and at times heightened by the nice weather in the summer (*n* = 46). This was particularly salient to those who lived in cramped houseshares who found lockdown especially challenging. Parents particularly highlighted the need to get their child out of the house and the importance for them to be active (*n* = 5).

“*After lockdown, my physical activity levels are instead dictated in large part by my toddler: I need to get him out of the house and do yoga with him for his physical and mental health primarily.”* **P52**

Mental health was a big motivator for a lot of people, with the increased stress brought on by lockdown and the pandemic overall (*n* = 53). While mental health had been a motivator for some before, it had become more of a primary concern for all and physical activity was seen as a key part of looking after one's mental well-being.

“*I find exercise really beneficial for combatting my anxiety. In lockdown, this is even more important to me than ever so I would say I am more motivated by mental health benefits than ever before.”* **P368**

##### Decreased Motivation

Other participants felt that lockdown had significantly decreased their motivation through the loss of social connection and the impact of self-isolating (*n* = 65). Being unable to attend their regular classes, their workout/running groups or sporting events made it more difficult for people to engage in physical activity. Moreover, the need to self-isolate, to help slow the spread of the virus, made people more conscious of partaking in physical activity that would involve going outside. This was also impacted by a general feeling of anxiety surrounding leaving the house, with some participants worried about leaving the house too often.

“*I'm really struggling to motivate myself to exercise on my own and only indoors. But it's not safe for me to exercise outside and with my friends so this is as good as it gets right now.”* **P11**

Participants noted a general change in motivation across the four follow-ups, the root of which ranged from the general stress of lockdown to tiredness and/or changes in mental health (*n* = 21). Overall, these changes made it more difficult for people to stay active and while for some these dips in motivation were temporary, others reported several weeks where their motivation levels impacted their ability to stay active.

“*Follow up 1: Lack of motivation on some days. Follow up 2: Motivation - I have found it hard to motivate myself some days. Weather - the weather hasn't been great the last week, which has lowered my motivation to go walking each day. Follow up 3: I've not had motivation to exercise on a number of days. I've also struggled to stay active with the heat of the last few days. Also, my mood hasn't made me want to exercise. Follow up 4: Motivation - have felt quite unmotivated on some days. Colder/wet weather has prevented me from walking some days. I've felt a little unwell on some days, which has meant I've not exercised as much as I normally would*.” **FP124**

##### Issues Faced Across Lockdown

Similarly, lack of space and equipment, remained an issue for people. These issues were particularly difficult for people in shared accommodation, either with family or housemates, where they did not have a space to use for exercise without disrupting others in the household (initial survey, *n* = 39 Follow ups, *n* = 14). In addition, issues with internet connectivity meant that even when people had developed new online routines, these were disrupted by external factors. This was coupled with a continued feeling of uneasiness with regards to exercising outside, with many participants worried that others were not appropriately social distancing in the places where they would go to exercise, for example, local parks (*n* = 14).

“*Follow up 1: I walk in the park at weekend, and in the afternoon the park is rammed with people and cyclists (not supposed to cycle in the park). The amount of people makes it difficult to keep social distancing.”* **FP59**

Finding the time for exercise during lockdown was also a recurring issue for people. Lack of time throughout the week made it difficult for people to maintain regular exercise routines; there were a few reasons for this lack of time including ongoing childcare commitments or increased socialising (online and in-person) as lockdown progressed. One of the biggest factors affecting people's time for exercise across the follow ups was pressure from work (*n* = 27). Work pressures came in the form of increased workloads, external pressures from employers or specific deadlines.

“*[My Motivation] hasn't changed, I just don't have the same opportunity to engage in it as I'm looking after the children all day every day every week.”* P141

Finally, a factor affecting many participants was illness and injury experienced during lockdown (n = 48). Some of these were incidental to lockdown, people getting a cold or toothache, however many experienced injuries related to their change in physical activity. People who had increased their sedentary time due to now working from home reported experiencing aches in the back and neck due to poor ergonomic setups and increased screen time. Moreover, there were several reports of sporting injuries being incurred due to the sudden change in activity type and intensity.

From Follow up 1: “*Some new right lower leg and ankle pain that comes on after jogging, not during. Usually, I only jog once a week but have increased this to twice weekly, as unable to go swimming.”* **FP54**

## Discussion

### The Reported Use of Physical Activity Technology During Lockdown

Firstly, from our results we see similar declines in physical activity due to lockdown for work and travel as well as an increase in sitting time, as reported in other works (34). However, we did not see a significant drop in people's reported vigorous work activity, plausibly largely due to the already low levels of vigorous work activity reported in our sample of predominantly desktop-based workers. Moreover, we saw an increase in participants' reported vigorous and moderate sport activity time, seemingly in response to the recognition of a decrease in other activity. Participants noted an increase in their awareness of the need to be active, both for the physical and mental health benefits that have been identified in prior works (1, 3, 19). Moreover, our findings suggest a change in people's motivations to be active. Participants noted a desire to be more active to combat the overall inactivity caused by lockdown and, for some, lockdown brought a reduction of the barriers people typically experience to being active, such as those shown in (6). Others found it a useful coping mechanism for dealing with lockdown and getting out of the house.

Physical activity technology use saw a significant increase during the lockdown, and the use of such technology remained stable thereafter among the participants who completed the four follow-up surveys. While many saw an increase, the high use of activity tracker use was already relatively high, with many people “rediscovering” their old activity trackers or changing the way they use them.

Activity tracking helped people stay aware of their activity levels, establish goals, and remember to move, as has been demonstrated outside of lockdown (20, 21) and reported by the companies such as Garmin and FitBit (41, 42). At the same time, technology appeared to influence the volume of time spent, specifically, on vigorous sport: activity trackers were found to significantly help people maintain vigorous sport levels in the first few weeks and fitness planning apps were linked to increased activity in the final follow-up week.

Use of online tutorials/classes and fitness apps was significantly more prevalent during lockdown than before lockdown. Online classes and groups allowed people to both continue accessing knowledge from trainers and stay connected with their exercise communities. This is in line with previous research exploring the use of virtual training platforms (23) and the importance of the social context of exercise (6, 30). Moreover, we observed that these different technologies also helped people develop accountability for staying active. This aspect of accessibility was particularly salient to those who had limited access to sophisticated equipment during lockdown and virtual activity gave them the tools to stay active as well as allowing them to get out of the house.

However, staying active in lockdown was not without its challenges. Many participants felt that their overall motivation had been reduced due to lockdown as well as experiencing an increase in tiredness, as well as a lack of time and a lack of resources necessary to stay engaged in physical activity, again this is a common factor seen outside of life disruptions such as lockdown (6). Moreover, people's sedentary time saw a significant increase due to lockdown, with physical activity technology use having no impact. Therefore, while many people replaced their day-to-day activity with vigorous exercise, there was a separate issue of increased sedentary time which in and of itself can lead to a number of serious health concerns (47). Several participants noted sports-related injuries resulting from the sudden change in activity levels/type. This could mean that while people were able to adapt their exercise routine, this was not always done safely. It could be that the technology used either encouraged overuse or did not highlight how to exercise without sustaining injury, with people lacking the contextual information, that has been shown to be important for the development of a physical activity routine (30).

### Keeping Active During Life Disruptions With the Use of Technology

From these findings, we have identified aspects that need to be addressed in the design of physical activity technology to help people keep active during life disruptions. While many of the responses dealt specific with issues related to the pandemic, the technological strategies people adopted to overcome these issues can tell us more generally how people stay resilient in staying active during life disruptions, as done in (17, 18). Some of these are overarching problem areas within PA technology, however there are also some specific barriers and opportunities which come into play during significant life disruptions as seen during the pandemic.

#### Known Issues in Physical Activity Technology Design

**Embedding behaviour change theory in the design of physical activity technologies**. Throughout lockdown people struggled to stay motivated to exercise. However, the aspects that participants in the current study found most useful for staying active were those that reduce the barriers to exercise and helped the develop a routine. It is well known that the majority of publicly available physical activity apps lack features drawing from the behaviour change theory (10, 11). Self-regulation is likely to always be an issue for people trying to stay active, but through effective behaviour change and through supporting people's habit formation, that barrier can be reduced. The ebbs and flow of people motivation across our follow-ups demonstrates a need for additional support for on-going motivation and development of habitual activity. If digital solutions are to be successful in aiding self-regulation, researchers need to create tools that support technology developers in embedding behaviour change theory into the design of physical activity apps.

**Supporting long term maintenance of physical activity routines**. While our results confirm prior findings on the impact of COVID-19 on global activity levels (34), we also found that participants were able to use technology to increase their vigorous activity in response to restrictions on other kinds of activity. Increased uptake in technology and awareness of inactivity means that, in the next few years, there is likely to be a pressing need for effective physical activity technology. There is an urgent requirement to examine how technology can help people maintain activity outside of lockdown and translate the routines developed in lockdown into long-term physical activity.

With the seeming success of activity tracking in helping people stay informed, create goals and use reminders to be active, there is room to explore how activity tracking could be used to form more long-lasting physical activity routines; as shown in prior works, through promoting further reflection on one's own data (20) or helping people break down their exercise goals (21). As we know from previous research there are several factors that indicate whether activity will be maintained in the long run (6, 48). Activity tracking can either build on positive factors, for example by creating social links to activity, as seen in some of the running apps, or it can help minimise the impact of negative factors, for example by addressing issues of self-belief/self-image through the use of body-centred feedback (49). This demonstrates further the need for research to explore how technology can be designed to support the formation of effective physical activity habits in the long-term, as previously identified (21, 31).

#### Opportunities Specific to Staying Active During Life Disruptions

**Personalisation of activity levels to support lower intensity activity and reduce injury**. Our findings also demonstrated that, despite some increases in activity, levels of moderate activity and levels of sedentary behaviour remained unaddressed. We know from previous works, that these kinds of activity are just as important to people's overall wellbeing as the more vigorous activity (47), however, it would appear that the majority of technologies primary focus on higher intensity activity. While there have been explorations of how technology can combat sedentary behaviour and lower intensity activity (50), it is necessary to highlight the importance of reducing sedentary time to people who use exercise technology. To add to this, the reports of injury are a cause for concern, as this too may lead to people abandoning a routine. This illustrates further the need for work looking at how technology can combat overactivity and encourage sustained exercise and understand the needs of the individual (30).

**Changing motivational needs caused by life disruptions**. A common problem seen in our study, was the impact of external factors caused by the pandemic which led to changing motivation to be physically active. This was in some ways positive, with people feeling more motivated to exercise, to get out of the house or to increase their step count, and in other ways negative, with people seeing dips in motivation due to low mood and difficulties in staying active. Moreover, we observed increased sedentariness and a lack of low intensity activity among our participants. This demonstrates a need for technology that both supports users through such changes in motivation and facilitates low intensity exercise and breaks from excessive sedentariness, which has been shown to be detrimental to people's well-being and health (47). Prior systems have been shown to effectively motivate breaks from sedentariness through prompting and exercise snacking (51, 52), which may be leveraged to support low intensity activity when motivation is low.

**Leveraging new digital social connections to support physical activity**. Using online training for physical activity was seen by people in lockdown as a way to stay connected with friends/exercise groups and to meet new people. These communities have been shown to have the potential to help support people during these life disruptions and create a semblance of normality in uncertain times (17). As we move into the future, maintaining these communities is likely to be more difficult, while remaining an important aspect of what keeps people engaged in physical activity (6). However, through closer consideration of the social aspect of activity, such as in (30), perhaps these connections can be maintained. As prior work has shown, maintaining online communities can be difficult (53). If the social connections that people made during lockdown (and importantly the accountability that came with them) are to be maintained, physical activity technology needs to adapt to people's schedules changing again as they return to work. Such technology should also accommodate for some of the barriers to activity that were reduced during lockdown and are likely to return post-lockdown. This is likely to come from leveraging the social connections made during lockdown, e.g., the personal connection between coach and trainee (30), as well as considering how people make connections that reach outside their exercise context, such as in (53). Another potential avenue for this would be employing the ethos of citizen science to support physical activity; this approach could help researchers both promote and study physical activity in a community setting (54).

### Limitations

The initial survey was launched at the end of May, ~2 months after the introduction of lockdown. This meant that, although we were able to ask participants about the changes they experienced when the lockdown was initially introduced, we were not able to track these changes in real-time. Moreover, we experienced attrition of participants across the four follow-up surveys, from *n* = 333 in the initial survey to a much smaller *n* = 84 sample of individuals who completed all five surveys, resulting in 74.77% attrition. This is not uncommon in the context of volunteer research participation, and while our participants were entered for a prize raffle, they did not receive payment for their time, as would typically be the case in traditional research participation. It should be noted that many citizen scientists are only interested in “dabbling” in citizen science initiatives (55) and projects routinely experience high attrition rates (56). Rotman et al., studied three long-term citizen science projects and found participant attrition to range between 80 and 95 percent (57); factors of particular importance for sustaining interest over time were relationships between volunteers and researchers, as well as between volunteers and their communities. This suggests that projects seeking to engage citizen science participants, over longer periods of time, could benefit from creating a community around the project, a factor that was missing in the current study.

Moreover, when sending out follow-up surveys, we allowed participants to respond within 7 days. While facilitating participant retention, this created variability in when participants responded at each follow-up stage. This may be unavoidable when obtaining responses from volunteer samples which, as discussed above, presents recruitment and retention challenges. Our initial findings are limited to individuals who decided to engage with and complete the initial survey, and the follow-up findings are limited to individuals who remained within the study for all five surveys. Investigating physical activity, we may have attracted those with greater experience of or interest in, physical activity. It is also possible that the enquiry into people's use of technology may have discouraged those with lower Internet literacy. In addition, this study did not explore differences in socio-economic background, generational differences or how different cultures responded to the pandemic. Though the aim in the current study was to give an overview of people's experience in staying active during the pandemic, there are likely more nuanced differences between different groups of people and their responses to such life disruptions.

Moreover, especially in the initial survey, but also in the follow-ups, we asked participants to self-report their activity and experiences. This retrospective collection of data may lead to issues surrounding with how they report their activity due to both issues surrounding recall and how they compare their prior experiences with how they felt during the survey. Similarly, there may be issues with participants reporting and their activity due to their inclusion in the study. As discussed above, our participants were already active prior to the study, but taking part in the study itself may have formed part of their motivation to stay active and to use their technology in certain ways.

## Conclusion

In this paper, we present a set of studies which examine how people used technology to support their physical activity during lockdown. Our surveys show both how people used technology to support their physical activity during lockdown and also how they perceived the benefits of such technology. We found that an increase in activity tracking use was associated with an increase in vigorous sports activity in response to lockdown, suggesting that tracking one's activity can help support people in maintaining an exercise routine even when their access to gyms and in-person classes is limited. We also saw a significant increase in people using online classes and video tutorials, which helped them learn new exercises and stay connected with others. Our qualitative responses show changes in people's motivation and a general increase in awareness of the importance of staying active due to the physical and mental health benefits. However, some people also experienced difficulties in staying active including reduced motivation and even injury. Additionally, while people were able to maintain a high level of vigorous activity, sedentary time was not impacted by technology use. From these results, we present directions for the future of physical activity technology research.

## Data Availability Statement

The raw data supporting the conclusions of this article will be made available by the authors, without undue reservation.

## Ethics Statement

The studies involving human participants were reviewed and approved by UCL. The patients/participants provided their written informed consent to participate in this study. Written informed consent was obtained from the individual(s) for the publication of any potentially identifiable images or data included in this article.

## Author Contributions

The study design, qualitative analysis and writing were led by JN. AR led both recruitment and the quantitative analysis. AC supervised the project and contributed guidance to each stage of the study design and implementation. AC and AR contributed to the writing. All authors contributed to the article and approved the submitted version.

## Funding

This work was supported by the EPSRC funded GetAMoveOn Network+ (EP/N027299/1).

## Conflict of Interest

The authors declare that the research was conducted in the absence of any commercial or financial relationships that could be construed as a potential conflict of interest.

## Publisher's Note

All claims expressed in this article are solely those of the authors and do not necessarily represent those of their affiliated organizations, or those of the publisher, the editors and the reviewers. Any product that may be evaluated in this article, or claim that may be made by its manufacturer, is not guaranteed or endorsed by the publisher.
